# Translational Signalling, Atrogenic and Myogenic Gene Expression during Unloading and Reloading of Skeletal Muscle in Myostatin-Deficient Mice

**DOI:** 10.1371/journal.pone.0094356

**Published:** 2014-04-09

**Authors:** Heather K. Smith, Kenneth G. Matthews, Jenny M. Oldham, Ferenc Jeanplong, Shelley J. Falconer, James J. Bass, Mônica Senna-Salerno, Jeremy W. Bracegirdle, Christopher D. McMahon

**Affiliations:** 1 Department of Sport and Exercise Science, University of Auckland, Auckland, New Zealand; 2 AgResearch Ltd., Ruakura Agricultural Centre, Hamilton, New Zealand; 3 Liggins Institute, University of Auckland, Auckland, New Zealand; University of Rome La Sapienza, Italy

## Abstract

Skeletal muscles of myostatin null (*Mstn*(−/−)) mice are more susceptible to atrophy during hind limb suspension (HS) than are muscles of wild-type mice. Here we sought to elucidate the mechanism for this susceptibility and to determine if *Mstn*(−/−) mice can regain muscle mass after HS. Male *Mstn*(−/−) and wild-type mice were subjected to 0, 2 or 7 days of HS or 7 days of HS followed by 1, 3 or 7 days of reloading (n = 6 per group). *Mstn*(−/−) mice lost more mass from muscles expressing the fast type IIb myofibres during HS and muscle mass was recovered in both genotypes after reloading for 7 days. Concentrations of MAFbx and MuRF1 mRNA, crucial ligases regulating the ubiquitin-proteasome system, but not MUSA1, a BMP-regulated ubiquitin ligase, were increased more in muscles of *Mstn*(−/−) mice, compared with wild-type mice, during HS and concentrations decreased in both genotypes during reloading. Similarly, concentrations of LC3b, Gabarapl1 and Atg4b, key effectors of the autophagy-lysosomal system, were increased further in muscles of *Mstn*(−/−) mice, compared with wild-type mice, during HS and decreased in both genotypes during reloading. There was a greater abundance of 4E-BP1 and more bound to eIF4E in muscles of *Mstn*(−/−) compared with wild-type mice (*P*<0.001). The ratio of phosphorylated to total eIF2α increased during HS and decreased during reloading, while the opposite pattern was observed for rpS6. Concentrations of myogenic regulatory factors (MyoD, Myf5 and myogenin) mRNA were increased during HS in muscles of *Mstn*(−/−) mice compared with controls (*P*<0.001). We attribute the susceptibility of skeletal muscles of *Mstn*(−/−) mice to atrophy during HS to an up- and downregulation, respectively, of the mechanisms regulating atrophy of myofibres and translation of mRNA. These processes are reversed during reloading to aid a faster rate of recovery of muscle mass in *Mstn*(−/−) mice.

## Introduction

Skeletal muscles atrophy when their normal workload, including habitual weight-bearing activity, is reduced. Hind limb suspension (HS) removes weight-bearing activity, unloading the muscle and thus causing disuse atrophy. Conversely, the reinstatement of weight-bearing, or reloading, leads to subsequent regrowth and recovery of the muscle [1]–[5]. In this regard, myostatin is a key gene regulating muscle mass. Mice lacking this gene (*Mstn*(−/−)) have a profound increase in growth of skeletal muscles that is attributed to both hyperplasia and hypertrophy of muscle fibers during development [6]. Postnatally, myostatin is also thought to play a role in regulating atrophy of skeletal muscle, the best example of which was shown in mice in which muscles were injected with cells that secreted high concentrations of myostatin [7]. In support, expression of myostatin was increased in muscles of patients confined to bed rest prior to surgery [8], and in muscles of rats after 17 days of micro-gravity in space-flight [9], and was either transiently or persistently increased during loss of muscle mass from rodents subjected to HS [10], [11]. Therefore, blockade of myostatin was proposed as a therapy to ameliorate the loss of muscle mass during various myopathies. However, at odds with that postulate, we have shown that the absence of myostatin rendered mice more susceptible to loss of muscle mass during HS, which suggests that myostatin is not required for muscle atrophy [12]. Rather, in the absence of myostatin, one or more of the processes regulating the mass of skeletal muscle may be compromised. Furthermore, the role of myostatin in the regrowth of disused skeletal muscle has yet to be considered.

While satellite cells are essential for post-natal growth [13], the regrowth and hypertrophy of adult skeletal muscle, including recovery after HS, can be achieved when satellite cells are absent, or depleted [14]–[18]. Therefore, recovery of lost muscle mass in adults is thought to be largely attributable to changes in the balance between synthesis and degradation of protein, without the need to recruit satellite cells. Synthesis of protein is primarily regulated by two key processes. The first is the engaging of mRNA with the ribosome, a process governed by 4E-BP1, a repressor of translation that binds the 5′ cap-end translation initiation factor eIF4E [19]. When phosphorylated, 4E-BP1 dissociates from eIF4E and enables assembly of the eIF4F complex (eIF4G and eIF4A bound to eIF4E). The eIF4F complex then unwinds the secondary structures at the 5′ termini of mRNA and binds the 40S ribosome by docking with eIF3 [20], [21]. The second process regulates the rate of translation initiation and is governed by the provision of GTP to eIF2⋅Met-tRNA_i_ by eIF2B. This ternary complex is then shuttled to the AUG codon on the 40S ribosome subunit [19], [22], [23]. Termination of translation is mediated by phosphorylation of the eIF2α subunit and subsequent dissociation of eIF2 from mRNA and the ribosome to be sequestered by eIF2B [19], [24]. Myostatin is thought to inhibit protein synthesis via inhibiting p70S6k, a cytosolic protein downstream of mTOR, that in turn regulates phosphorylation of 4E-BP1 and rpS6 [25], [26].

Atrophy of skeletal muscle is largely regulated by the ubiquitin-proteasome and the autophagy-lysosomal systems. In the ubiquitin-proteasome pathway, the key ligases that target proteins for degradation are MAFbx (also called atrogin-1) and MuRF1 [27]–[29]. MAFbx has also been identified as a suppressor of protein synthesis [30]. Recently, expression of both genes was shown to be unchanged with denervation-induced atrophy of skeletal muscles [31]. Instead, a new ubiquitin ligase, MUSA1, was identified and shown to be causally related to the atrophy and regulated by the BMP family of proteins [31]. It is unclear at present whether or not myostatin regulates atrophy of skeletal muscles, with evidence suggesting a role in both increasing and decreasing the expression of MAFbx and MuRF1 in myoblasts [25], [32]. The autophagy-lysosomal pathway plays a role in the removal of unfolded, toxic and long-lived proteins and organelles after breakdown and is mediated by a number of genes, key among which are MAP1LC3 (LC3), Gabarapl1 and Atg [33]–[36].

Skeletal muscles are composed of fast- and slow-twitch myofibres. Myosin is a major contractile protein in muscle. There are four myosin heavy chain isoforms (MyHC) expressed in limb skeletal muscles, designated I, IIa, IIx and IIb [37]–[40]. MyHC type I is a slow-twitch isoform, type IIa is an intermediate twitch, and types IIb and IIx are fast-twitch. It is now well established that *Mstn*(−/−) mice have more type IIb myofibres than wild-type mice [41], [42] and it remains unclear whether all myofibre types are equally susceptible to HS-induced atrophy [5]. Associated with myofibre type is the expression of myogenic regulatory factors (MRFs), which are bHLH transcription factors that bind to E-boxes to regulate the transcription of target genes [43]–[45]. Specifically, MyoD and Myf5 regulate specification and proliferation, while myogenin and MRF4 regulate differentiation of myoblasts [43], [46]–[49]. However, in adult skeletal muscle, these factors are thought to regulate the balance between fast- and slow-twitch myofibres, wherein MyoD is predominantly expressed in type IIx and IIb myofibres, while myogenin is predominantly expressed in type I myofibres [50], [51]. Expression of MRFs was reported to increase during HS [52] and, therefore, given that muscles of *Mstn*(−/−) mice have a higher proportion of fast-twitch fibres [53], the expression of MRFs might be expected to increase to a greater extent in muscles of *Mstn*(−/−), when compared with wild-type mice.

Given that *Mstn*(−/−) mice are more susceptible to loss of muscle mass during HS [12], we hypothesised that one or more of the processes regulating the balance between protein synthesis and degradation are disrupted. It is also unclear if *Mstn*(−/−) mice will regain muscle lost during HS to the same extent as that of wild-type mice when muscles are reloaded. If true, then induced changes to these regulatory processes during HS might be reversed during reloading. To test this hypothesis, we subjected wild-type and *Mstn*(−/−) mice to up to seven days of HS to induce atrophy of skeletal muscle, or to seven days of HS followed by up to seven days of reloading. We measured the changes in body and muscle mass and the abundance of MyHC protein isoforms and cross-sectional area of myofibres, key regulatory factors associated with degradation (ubiquitin-proteasome and autophagy-lysosomal systems), key regulatory steps associated with the synthesis of proteins and the myogenic regulatory factors associated with maintaining the composition of fast- and slow-twitch myofibres.

## Methods

### Ethics Statement

The HS procedure has been described previously [12] and the study was approved by the Ruakura Animal Ethics Committee, Hamilton, New Zealand.

### Animals

Thirty-six male wild-type (C57) and 36 male *Mstn*(−/−) mice (16–18 weeks of age) were obtained from the Small Animal Colony at the Ruakura Agricultural Centre. Generation of *Mstn*(−/−) mice has previously been described [6]. We obtained a breeding pair of these mice as a gift from S-J Lee (Johns Hopkins School of Medicine, Baltimore, MD, USA).

### Experimental Design

Muscles were unloaded by HS for 0, 2 or 7 days, or unloaded by HS for 7 days, then reloaded by the reinstatement of weight-bearing and killed on days 8, 10 and 14 (n = 6 per group). Mice were killed via CO_2_ asphyxiation followed by cervical dislocation and care was taken to ensure that mice did not re-load before death. At death, the *Biceps femoris,* lateral *gastrocnemius, Quadriceps femoris (Quad), soleus, plantaris* and *Extensor digitorum longus (EDL)* muscles were excised, weighed, then snap frozen in liquid nitrogen and stored at –80°C for subsequent analysis. Given our previous results showing that predominantly fast-twitch muscles of *Mstn*(−/−) mice lose mass with HS [12] and the experimental constraints presented by the mass of the muscles (particularly the *soleus*), we performed a number of assays on *gastrocnemius* and *B. femoris* muscles. Unfortunately, not all experiments could be performed on the same muscle due to the small size of mouse muscles.

### Total RNA Extraction and Complementary DNA Synthesis

Total RNA was extracted from *B. femoris* muscles using Trizol reagent (Invitrogen, California, USA) according to the manufacturer’s protocol. The RNA was resuspended in DEPC-treated water and the concentration was quantified by nanodrop. Complementary DNA (cDNA) was reverse transcribed from 2 μg total RNA using qSCRIPT (Quanta BioSciences, Maryland, USA), following the manufacturer’s instructions.

### Quantitative (q) PCR

Each reverse transcribed (RT) reaction was diluted to 1∶10 and qPCR was carried out using a Lightcycler 2.0 (Roche Diagnostics, Germany) and Roche Faststart DNA master PLUS SYBR Green I mix (Roche Diagnostics). The primers used to quantify MyoD, Myf5, Myogenin, MAFbx, MuRF1, MUSA1, Atg4b, Gabarapl1 and MAP1lc3b (LC3b) were designed to span across exons and are: MyoD (134 bp product): forward 5′–CGCGCTCCAACTGCTCTGAT–3′; reverse 5′– CACAGCCGCACTCTTCCCT –3′; Myf5 (352 bp product): forward 5′–TGCCATCCGCTACATTGAGAG –3′; reverse 5′– CCGGGGTAGCAGGCTGTGAGTTG –3′; Myogenin (111 bp product): forward 5′–GCACTGGAGTTCGGTCCCAA –3′; reverse 5′- ATCCTCCACCGTGATGCTG –3′; MAFbx (215 bp product): forward 5′–AGCCAAGAAGAGAAAGAAAGAC –3′; reverse 5′- GACTTTGCTATCAGCTCCAAC –3′; MuRF1 (239 bp product): forward 5′–GACTGAGCTGAGTAACTGCATCTC –3′; reverse 5′– GGATCAGAGCCTCGATGAAG –3′; MUSA1 (189 bp product): forward 5′-CCGGCTAATGAGGGACGTTT -3′; reverse 5′- TGGTCAGCCATGCTCAGGAT -3′; Atg4b (246 bp product): forward 5′-ATTGCTGTGGGGTTTTTCTG- 3′; reverse 5′-AACCCCAGGATTTTCAGAGG-3′; Gabarapl1 (192 bp product): forward 5′-CATCGTGGAGAAGGCTCCTA-3′; reverse 5′-ATACAGCTGGCCCATGGTAG-3′ and LC3b (274 bp product): forward 5′-AAGGGAAGTGATCGTCGCCGGAGT-3′; reverse 5′-TGAGCTGCAAGCGCCGTCTGATT-3′. The reaction conditions were as follows: Denature for 5 min at 95°C, followed by 45 cycles of 95°C for 5 s, 60°C for 10 s and 72°C for 20 s. Standard curves were established by serial dilution of a pool of cDNA obtained from each sample. Results for each sample were normalised to the concentration of cDNA in the RT samples [54].

### Western Blot Analyses

A 150 mg sample of lateral *gastrocnemius* muscle was homogenised in 1 ml of lysis buffer (10 mM Hepes, 10 mM KCl, 1.5 mM MgCl, pH 7.9) with 0.5% IGEPAL detergent (Sigma, MO, USA) and an enzyme inhibitor (Complete, Roche Diagnostics). Samples were homogenized on ice, then centrifuged at 11,000×*g* for 10 min. Supernatant was recovered, mixed with Laemmli loading buffer [55], boiled for 5 min, then stored at –20°C until analysis. The protein concentration of the supernatant was determined using the bicinchoninic acid assay (Sigma-Aldrich NZ, Auckland, NZ).

Twenty micrograms of protein from each muscle sample was loaded and separated in a 10% SDS-polyacrylamide gel (eIF2α, rpS6) or a 15% SDS-polyacrylamide gel (4E-BP1), then transferred to a nitrocellulose membrane at 30 V overnight. Membranes were stained with Ponceau S to verify transfer of protein. They were then blocked in a 0.05 M Tris buffered saline with 0.05% (v/v) Tween 20 (TBST, pH 7.6) supplemented with 1% polyvinylpyrrolidone, 1% polyethylene glycol and 0.3% BSA for 2 h at room temperature and incubated overnight with one of rabbit anti-4E-BP1 (1∶1000, #R113, Santa Cruz Biotechnology Inc., Tx, USA), rabbit anti-phospho-4E-BP1 (1∶3000, Thr37/46, #236B4, Cell Signaling Technology Inc, MA, USA), rabbit anti-eIF2α (1∶1000, #11386, Santa Cruz Biotechnology Inc.), rabbit anti-phospho eIF2α (1∶2000, Ser51, #ab4837, Abcam, Cambridge, UK), rabbit anti-rpS6 (1∶1000, #5G10, Cell Signaling Technology Inc), rabbit anti-phospho-rpS6 (1∶500, Ser235/236, # 22118, Cell Signaling Technology Inc), or rabbit anti-actin (1∶5000, #A2066, Sigma-AldrichNZ, Auckland, NZ) to assess uniformity of loading. After incubating with the primary antibody, membranes were then washed in TBST, incubated with HRP-conjugated goat anti-rabbit secondary antibody (# PO448, DakoCytomation, Med-Bio, Christchurch, New Zealand) at 1∶5,000 for 2 h, then washed again and developed with enhanced chemiluminescence. The optical densities of each immunoreactive band were captured with a densitometer (GS 800, Bio-Rad Laboratories (NZ) Pty Ltd, Auckland, NZ) and analysed using Quantity One software (Bio-Rad Laboratories (NZ) Pty Ltd).

### m^7^GTP-sepharose Chromatography

To verify that the phosphorylation status of 4E-BP1 reflected binding to eIF4E, we used m^7^GTP-sepharose 4B resin to isolate and assess the bound state of these proteins for a loaded (day 0) and unloaded (day 2) comparison as previously described [56], [57]. Briefly, the m^7^GTP-sepharose 4B resin (GE Healthcare Ltd, Auckland, NZ), was washed twice with lysis buffer (described above) and 80 μl of a 50% slurry was mixed with 300 μg of supernatant from homogenised *gastrocnemius* muscle and allowed to incubate overnight at 4°C. After centrifugation at 13,000×*g* and three washes in 1 ml lysis buffer, the bound material was re-suspended in an equal volume of 2x Laemmli loading buffer. Western blotting was performed by loading 30 μl of sample on 10% (eIF4E) or 15% (4E-BP1) SDS-PAGE gels. Membranes were blocked as described earlier and 4E-BP1 was detected by incubating with rabbit anti-4E-BP1 antibody (1∶2000, Cell Signaling Technology Inc), while eIF4E was detected by incubating with mouse monoclonal anti-eIF4E antibody (1∶2000, #sc9976, Santa Cruz Biotechnology Inc) overnight. Detection was as described earlier.

### Assessment of Myosin Heavy Chain Isoforms

Changes in the composition of MyHC in skeletal muscle were determined electrophoretically using a modification of techniques described previously. Briefly, 150 mg of *B. femoris* was homogenised in 1 ml of lysis buffer (above) and 100 μl of lysate was added to 200 μl of an 8 M urea/2 M thiourea buffer. This buffer was desalted using the AG501-X8 mixed-bed resin (12.5 g/50 ml, Bio-Rad Laboratories), before adding dithiothreitol (75 mM), SDS (3% w/v), bromophenol blue (0.004% w/v) and Tris base (0.05 M, pH 6.8) [58], [59]. A 4% stacking gel (50∶1 acrylamide:bis) was made with 30% (v/v) glycerol and an 8% separating gel (50∶1 acrylamide:bis) was made with 35% (v/v) glycerol [38], [39], [60]. The lower running buffer consisted of 0.05 M Tris (base), 75 mM glycine, and 0.05% w/v SDS. The upper running buffer was at six times the concentration of the lower running buffer and β-mercaptoethanol was added at a final concentration of 0.08% v/v. Gels were run at 70 V for 40 h at 4°C, stained with Coomassie Blue (G450), and the optical density of each MyHC band determined by densitometry (GS 800 scanner coupled with Quantity One Software, BioRad Laboratories) and expressed as a percent of the total for each lane. We have found that this method gives cleaner band separation, particularly for murine MyHC, than does the traditional Laemmli buffer method [38].

### Muscle Fibre Size

The mean muscle fiber cross-sectional area was determined from transverse cryosections (6 μm) cut from the mid-belly of *gastrocnemius* muscles. Sections were labelled using immunohistochemistry to identify the basal lamina of muscle fibers. Sections were post-fixed in 10% (v/v) formalin for 5 min then washed and incubated with a rabbit polyclonal anti-laminin (#Z0097, DakoCytomation, 1∶100 in phosphate buffered saline (PBS) with 0.05% (v/v) Tween 20) overnight at 4°C. A biotinylated donkey anti-rabbit (#RPN1004, GE Healthcare Ltd, Auckland, NZ) secondary antibody was applied (1∶300) at room temperature for 30 min, followed by streptavidin Alexafluor 488 (Molecular Probes, Life Technologies NZ Ltd, Auckland, NZ) at 1∶400 for 30 min. To identify nuclei, DAPI (1∶1000 in PBS, 5 min) was used. Non-overlapping images of the entire muscle section were obtained using a microscope (DMI6000B) with motorized stage, digital color camera (DFC300) and capture software (AF6000) (Leica Microsystems, Wetzlar, Germany). Composite (tiled) images of the section were created and analysed. The mean fibre cross-sectional area was determined by manual tracing of the perimeter of clusters of contiguous fibers in artefact-free regions distributed across the section (mean  = 275 fibers per muscle) using commercial imaging software (ImagePro Plus, Media Cybernetics Inc, Rockville, MD).

### Statistical Analysis

The wet mass of muscles was expressed relative to the initial body mass on d0. Data were subjected to analysis of variance using GenStat version 13 (VSN International Ltd) with factors of genotype (*Mstn*(−/−) or wild-type), day and their interaction included in the model statement. Post-hoc multiple comparisons were performed using the method of Tukey [61]. Data are presented as means and the standard error of the mean (sem).

## Results

**Figure 1:**
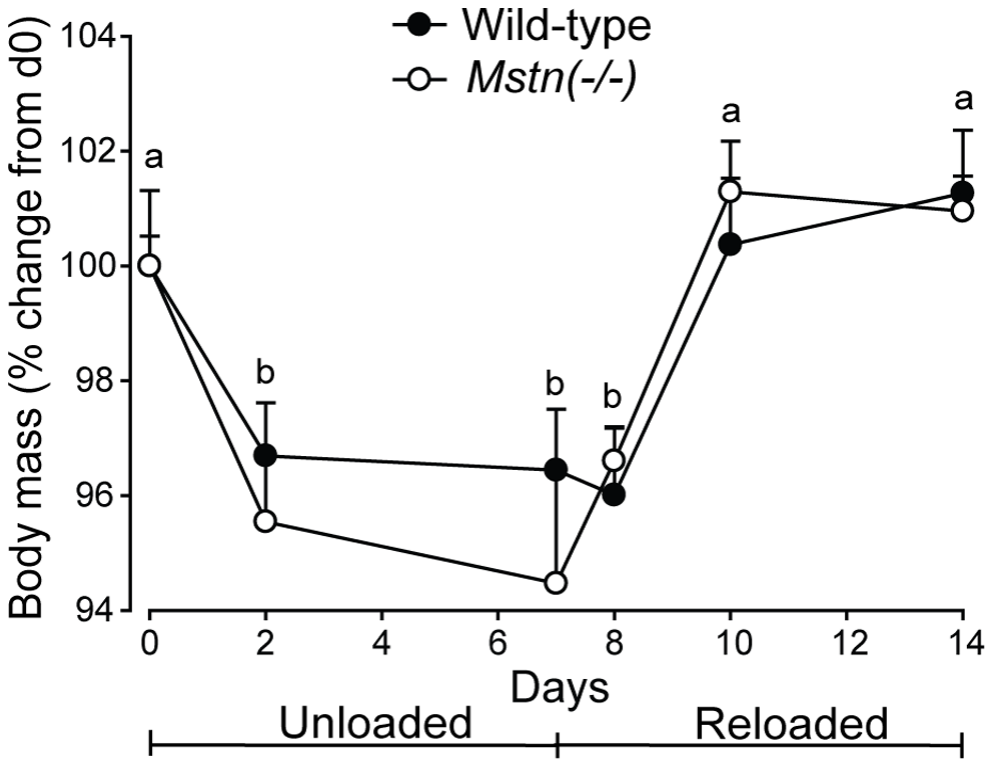
Body mass (mean+sem) for *Mstn*(−/−) and wild-type mice during seven days of unloading followed by seven days of reloading (n = 6 per genotype and day). Unlike letters denote significant differences (*P*<0.05) across days (independent of genotype).

**Figure 2:**
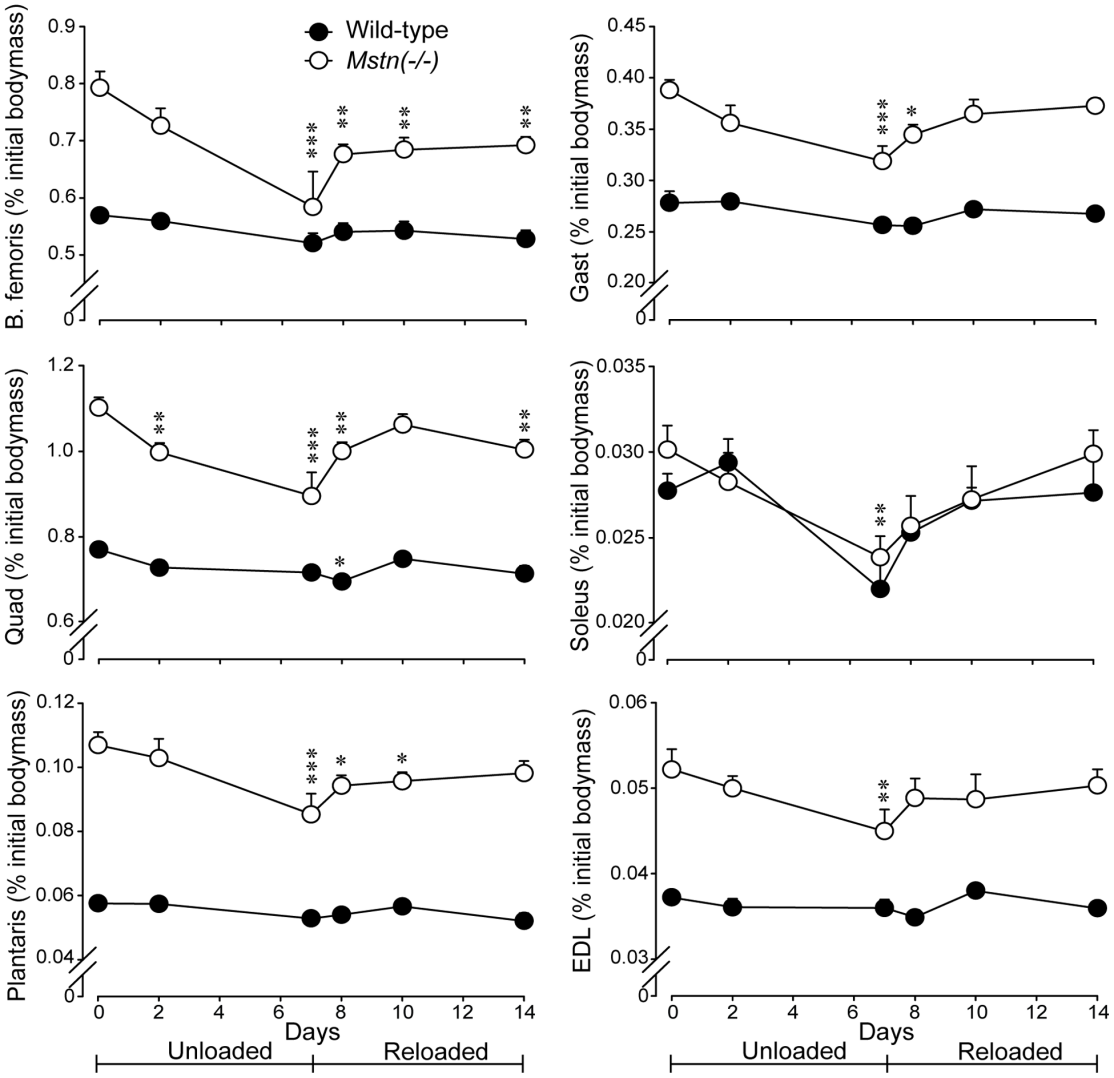
Muscle mass (mean+sem) expressed as a percent of the initial body mass at d0 for *Mstn*(−/−) and wild-type mice at days 0, 2 and 7 of unloading and days 8, 10 and 14 of reloading. The asterisks denote differences from d0 within genotype at the days noted (*P<0.05, **P<0.01 and ***P<0.001). The asterisks (**P<0.01) in the data for soleus indicates that muscle mass has been lost equally from both genotypes at d7. EDL = *Extensor digitorum longus*, Gast = *gastrocnemius*, Quad = *Quadriceps femoris*.

Mice of both genotypes lost body mass during HS and regained body mass to similar extents during reloading (*P*<0.001) (Figure 1). All muscles collected from *Mstn*(−/−) mice lost muscle mass during HS, while only the soleus of wild-type mice lost mass during HS. The lost mass of muscles from *Mstn*(−/−) mice was largely restored after 7 d of reloading. However, the mass of the *B. femoris* and *Quad* muscles of *Mstn*(−/−) were not fully recovered by d7 of reloading (Figure 2). The relative loss (∼20%, *P*<0.01) and subsequent gain (∼25%, *P*<0.001) of muscle mass in the *soleus*, which is composed of myofibres expressing only type I and IIa MyHC protein, were similar between genotypes. In contrast, *Mstn*(−/−) mice had greater losses and subsequent gains in the mass of muscles of predominantly type IIx and IIb MyHC-expressing myofibres than did wild-type mice (Figure 2).

**Figure 3:**
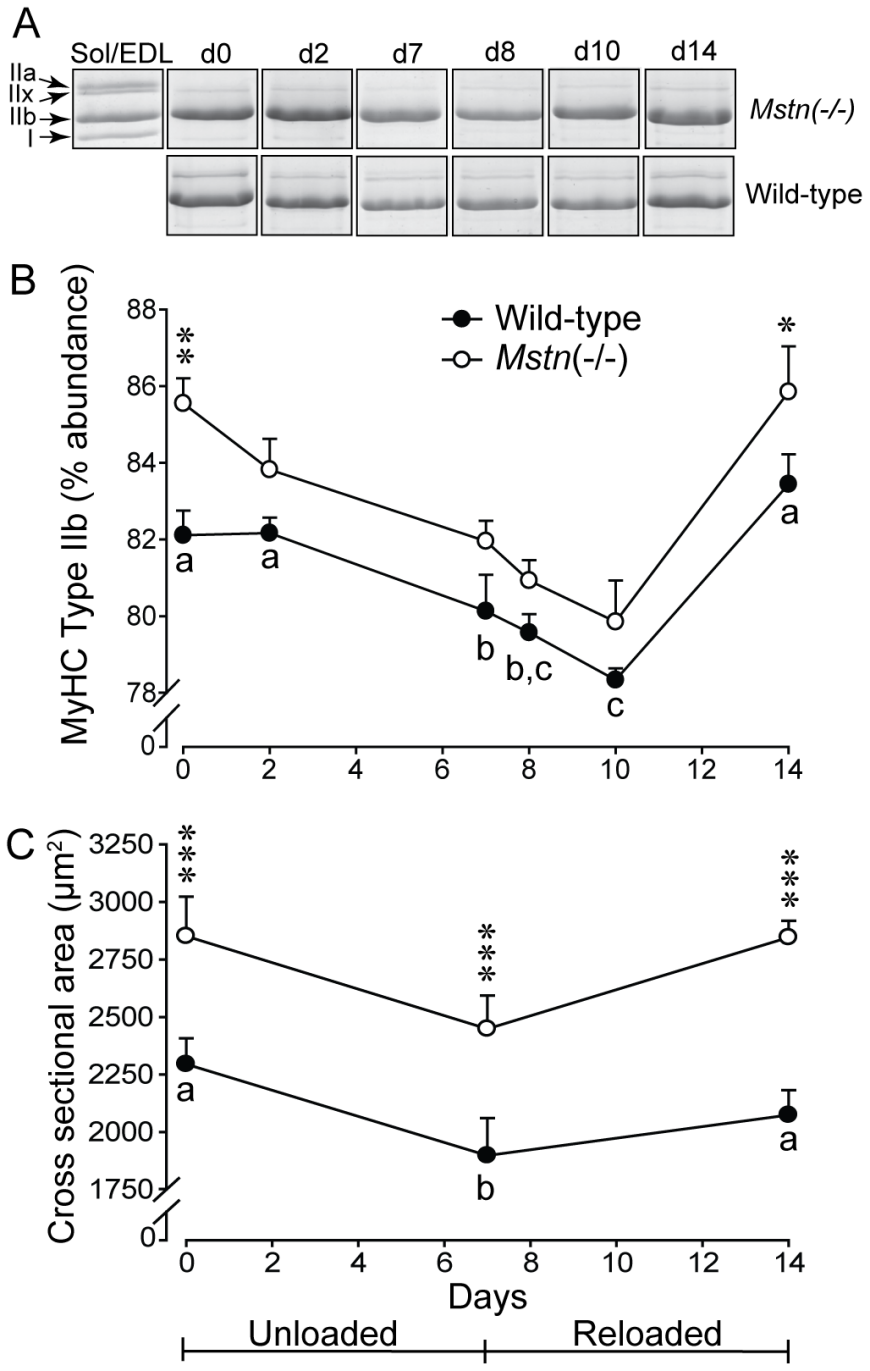
Changes in the composition of MyHC and the cross-sectional area of muscle fibres. (A) Representative gels stained with coomassie blue showing the myosin heavy chain (MyHC) protein isoforms in the *B. femoris* muscle of *Mstn*(−/−) and wild-type mice during seven days of unloading and seven days of reloading. A mixture of 1∶1 *soleus* and *Extensor digitorum longus* served as a ladder. (B) The change (mean+sem) in the relative abundance of type IIb MyHC protein in the *B. femoris* muscles is shown for *Mstn*(−/−) and wild-type mice (n = 6 per genotype and day) during seven days of unloading and seven days of reloading. There were main effects of day (*P*<0.001) and genotype (*P*<0.01), but no day×genotype interaction. Asterisks denote significant differences between genotypes (*P<0.05, **P<0.01). (C) Cross-sectional area (mean+sem) of myofibres in the *gastrocnemius* muscle of *Mstn*(−/−) and wild-type mice during seven days of unloading and seven days of reloading. The cross-sectional area was significantly reduced in both genotypes at d7 (P<0.05), before being restored to pre-unloading areas at d14. Asterisks denote significant differences between genotypes (***P<0.001). Unlike letters denote significant differences (*P*<0.05) across days (independent of genotype).

Electrophoretic separation of MyHC protein isoforms showed that *Mstn*(−/−) mice had a greater proportion of type IIb MyHC in the *B. femoris* muscles (85.5±0.6%) than wild-type mice (82.1±0.6%) before HS (*P*<0.01) (Figure 3A and 3B). However, there was a greater loss of type IIb MyHC in *Mstn*(−/−) compared with wild-type mice (effects of day *P*<0.001 and genotype *P*<0.01, no significant interaction). The proportion of type IIb MyHC was restored in both genotypes at day 14. There was no change in the proportion of type IIa or IIx MyHC while the percentage of type I MyHC was inversely proportional to that of type IIb, which is an artefact of expressing each isoform as a percentage of the total MyHC (data not shown). In conjunction, the cross-sectional area of myofibres was reduced in *gastrocnemius* muscles of both genotypes during HS and was restored after 7 d of reloading (Figure 3C).

**Figure 4:**
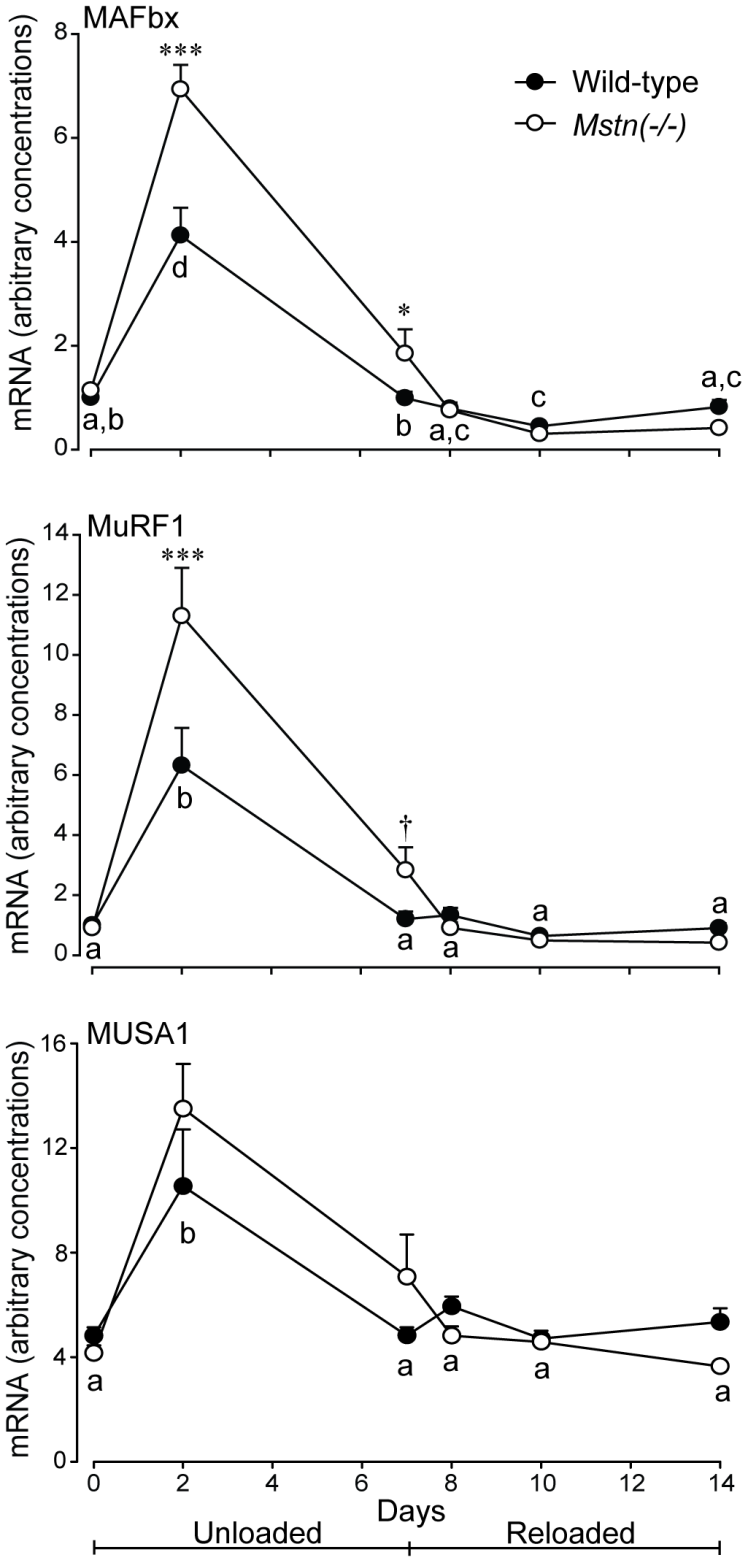
Arbitrary concentrations (mean+sem) of MAFbx, MuRF1 and MUSA1 mRNA in the *B. femoris* muscles of *Mstn*(−/−) and wild-type mice during seven days of unloading and seven days of reloading (n = 6 per genotype and day). The dagger and asterisks denote differences between genotypes on days shown (^†^ *P*<0.10, *P<0.05, ***P<0.01). Unlike letters denote significant differences (*P*<0.05) across days (independent of genotype).

Concentrations of MAFbx, MuRF1 and MUSA1 mRNA were not different in the *B. femoris* muscles between genotypes at day 0, increased dramatically in both genotypes at day 2, then declined to day 10 and remained low thereafter (Figure 4). In addition, the concentrations of MAFbx and MuRF1 were higher in muscles of *Mstn*(−/−) mice at day 2 (*P*<0.001) and were either higher (*P*<0.05, MAFbx), or tended to be higher (*P*<0.1, MuRF1) at day 7 compared to muscles of wild-type mice. In contrast, concentrations of MUSA1 were not different between the two genotypes throughout the unloading and reloading phases of the study.

**Figure 5:**
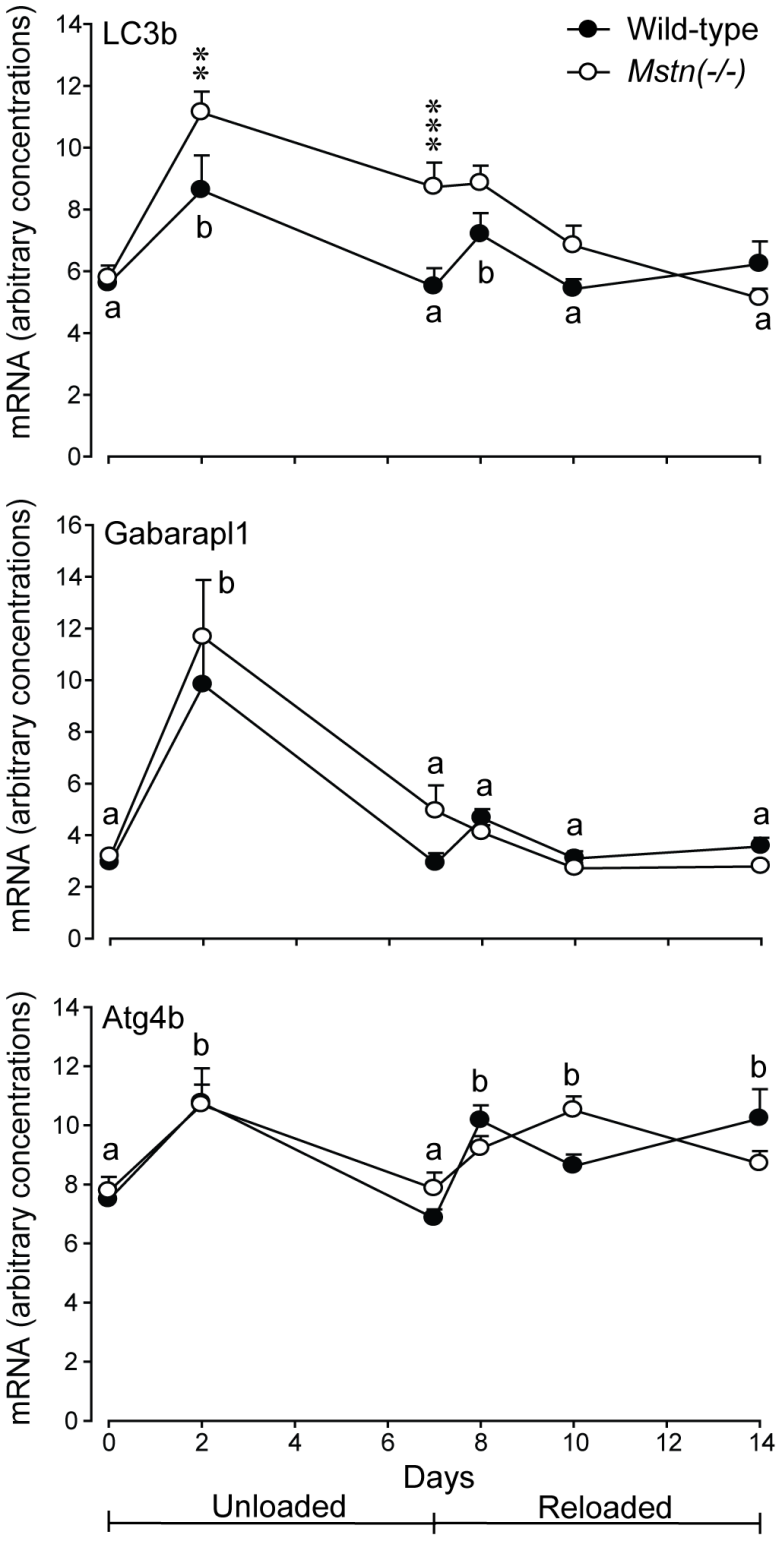
Arbitrary concentrations (mean+sem) of LC3b, Gabarapl1 and Atg4b mRNA in the *B. femoris* muscles of *Mstn*(−/−) and wild-type mice during seven days of unloading and seven days of reloading (n = 6 per genotype and day). The asterisks denote differences between genotypes on days shown (**P<0.01, ***P<0.01). Unlike letters denote significant differences (*P*<0.05) across days (independent of genotype).

Concentrations of LC3b, Gabarapl1 and Atg4b mRNA were not different between genotypes at day 0, and increased to a peak at day 2 of HS, before declining (Figure 5). LC3b mRNA continued to decline for the remainder of the study, Gabarapl1 mRNA reached pre-HS concentrations at day 8 and were unchanged during reloading, whereas Atg4b mRNA increased again from day 7 and remained so during the reloading phase (Figure 5). In addition, concentrations of LC3b mRNA were greater in the *B. femoris* muscles of *Mstn*(−/−) mice at day 2 (*P*<0.001, major effect of genotype) and remained higher than wild-type mice for most of the study (Figure 5).

**Figure 6:**
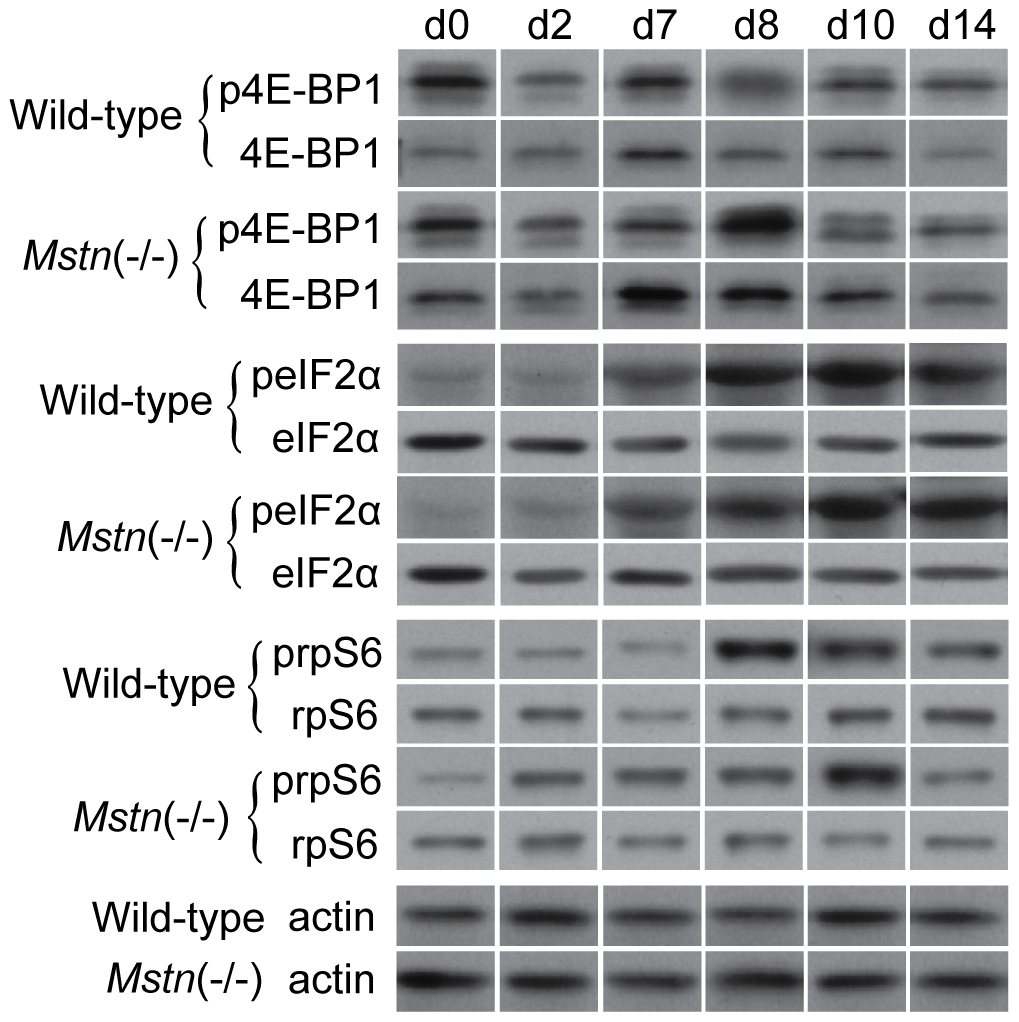
Representative western blots of phosphorylated (p) and total 4E-BP1, eIF2α, rpS6 and actin (loading control) in *gastrocnemius* muscles of *Mstn*(−/−) and wild-type mice at days 0, 2 and 7 of unloading and days 8, 10 and 14 of reloading.

**Figure 7:**
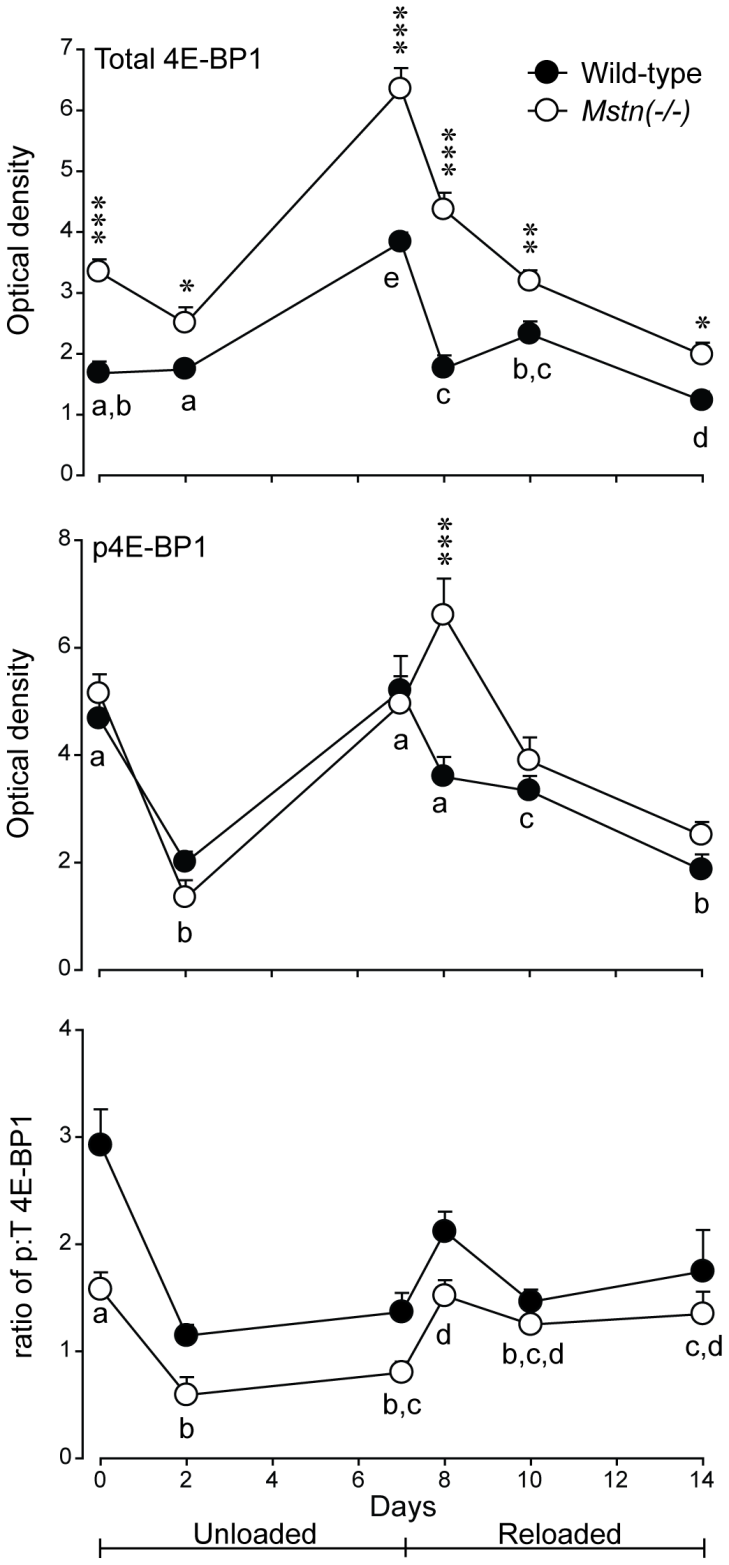
Abundance (mean+sem) of total (T) 4E-BP1, phosphorylated (p) 4E-BP1 and the ratio of p:T 4E-BP1 in *gastrocnemius* muscles of *Mstn*(−/−) and wild-type mice (n = 6 per genotype and day) during seven days of unloading and seven days of reloading. Asterisks denote significant differences between genotypes at the days indicated (*P<0.05, **P<0.01, ***P<0.001). Unlike letters denote significant differences (*P*<0.05) across days (independent of genotype).

**Figure 8:**
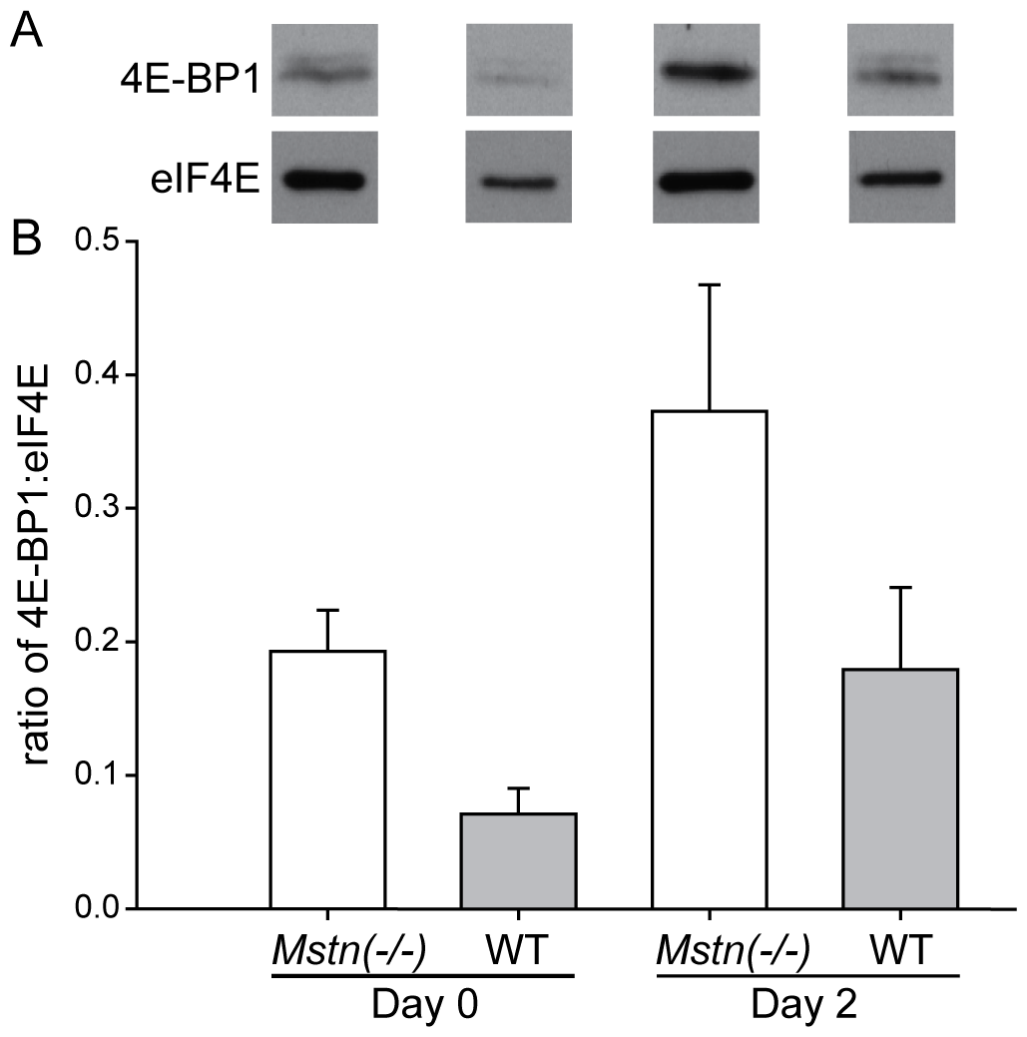
The amount of 4E-BP1 bound to eIF4E. (A) Representative western blots of 4E-BP1 bound to eIF4E and (B) the ratio (mean+sem) of 4E-BP1 bound to isolated eIF4E purified by an m^7^GTP-sepharose pull-down assay in the *gastrocnemius* muscle of *Mstn*(−/−) and wild-type mice (n = 6 per genotype and day) before and after two days of unloading. There were main effects of day (*P*<0.05) and genotype (*P*<0.05), but no day × genotype interaction.

Representative western blots are shown for total and phosphorylated 4E-BP1, eIF2α, rpS6 and actin (loading control) in Figure 6. There was a greater abundance of total 4E-BP1 overall in muscles of *Mstn*(−/−) mice compared with those in wild-type mice (*P*<0.001). The abundance of total 4E-BP1 increased to d7, before declining below pre-HS values at d14 (Figure 7). The abundance of phosphorylated 4E-BP1 was significantly different between the two genotypes at d8 only, was reduced in both genotypes at d2, increased at d7 (d8 in *Mstn*(−/−)) and then declined for the remainder of reloading. In contrast, the ratio of phosphorylated to total 4E-BP1 was lower before HS and remained lower throughout the HS and reloading phases in the *gastrocnemius* muscles of *Mstn*(−/−) compared with wild-type mice (Figure 7). Most notable was a decline in the ratio during HS in both genotypes. A subsequent increase at day 8 and gradual recovery during reloading was evident (effects of day *P*<0.001 and genotype *P*<0.001 alone, no significant interaction). To confirm that a reduction in phosphorylation of 4E-BP1 reflected increased binding to eIF4E, we used m^7^GTP sepharose to specifically isolate and enrich 4E-BP1 bound to eIF4E for days 0 and 2 for comparison between a loaded and an unloaded state. Consistent with the greater abundance of total 4E-BP1, which gave rise to the lower ratio of phosphorylated to total 4E-BP1 (Figure 7), there was more 4E-BP1 bound to eIF4E in muscles of *Mstn*(−/−) compared with wild-type mice before HS and this ratio increased after 2 days of HS in both genotypes (effects of day *P*<0.05 and genotype *P*<0.05 alone, no significant interaction) (Figure 8).

**Figure 9:**
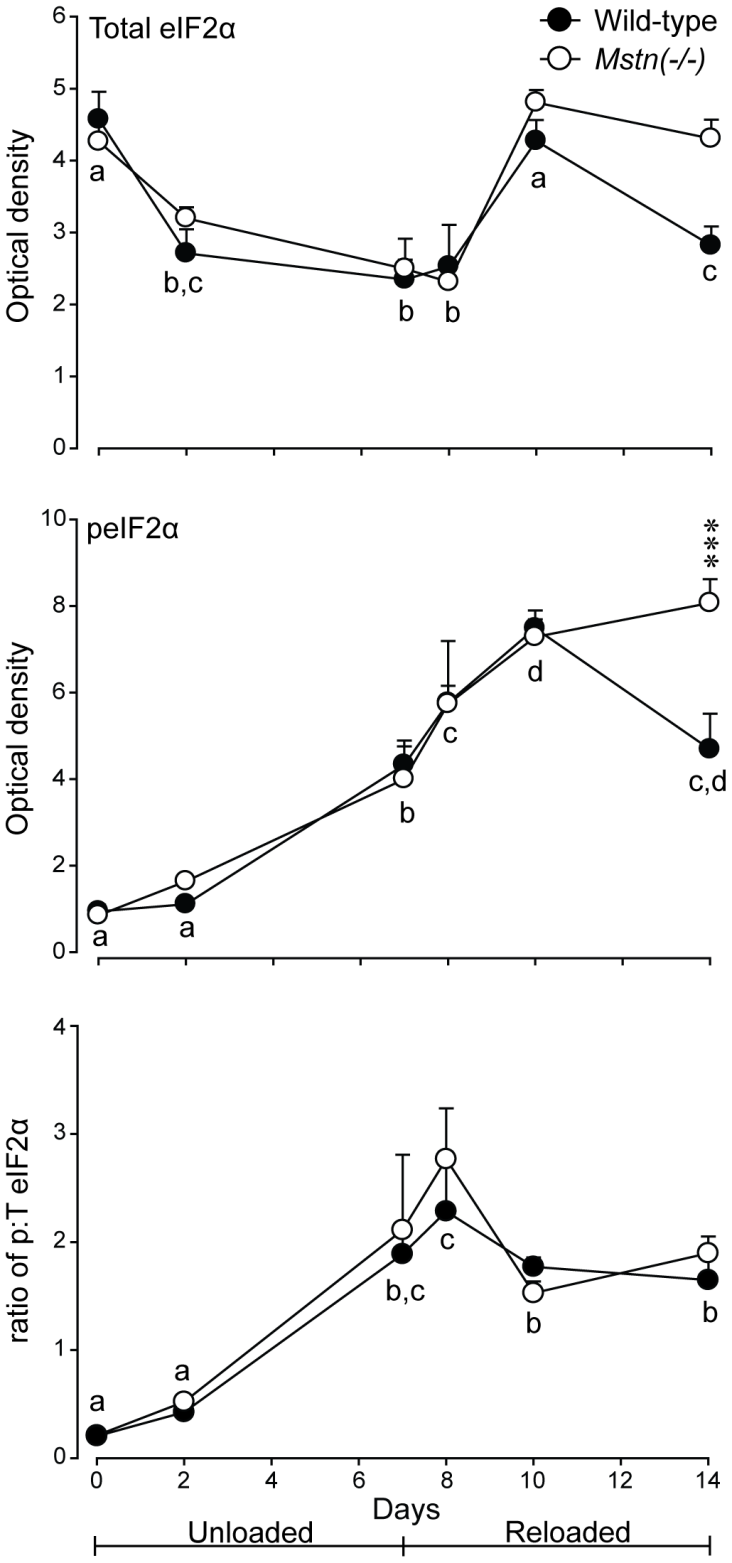
Abundance (mean+sem) of total (T) eIF2α, phosphorylated (p) eIF2α and the ratio of p:T eIF2α in *gastrocnemius* muscles of *Mstn*(−/−) and wild-type mice (n = 6 per genotype and day) during seven days of unloading and seven days of reloading. Asterisks denote significant differences between genotypes at the days indicated (***P<0.001). Unlike letters denote significant differences (*P*<0.05) across days (independent of genotype).

The abundance of total eIF2α was not different between the genotypes. It was reduced during HS and was restored at d10 (three days of reloading), before being reduced again at d14 (Figure 9). The abundance of phosphorylated eIF2α was not different between the genotypes and increased during HS and the first three days of reloading, then declined in muscles of wild-type mice at d14 (Figure 9). As with 4E-BP1, the ratio of phosphorylated to total eIF2α was more stable than the abundance of either the total or phosphorylated proteins and did not differ between genotypes. The ratio increased (*P*<0.001) during HS and remained elevated during reloading (Figure 9).

**Figure 10:**
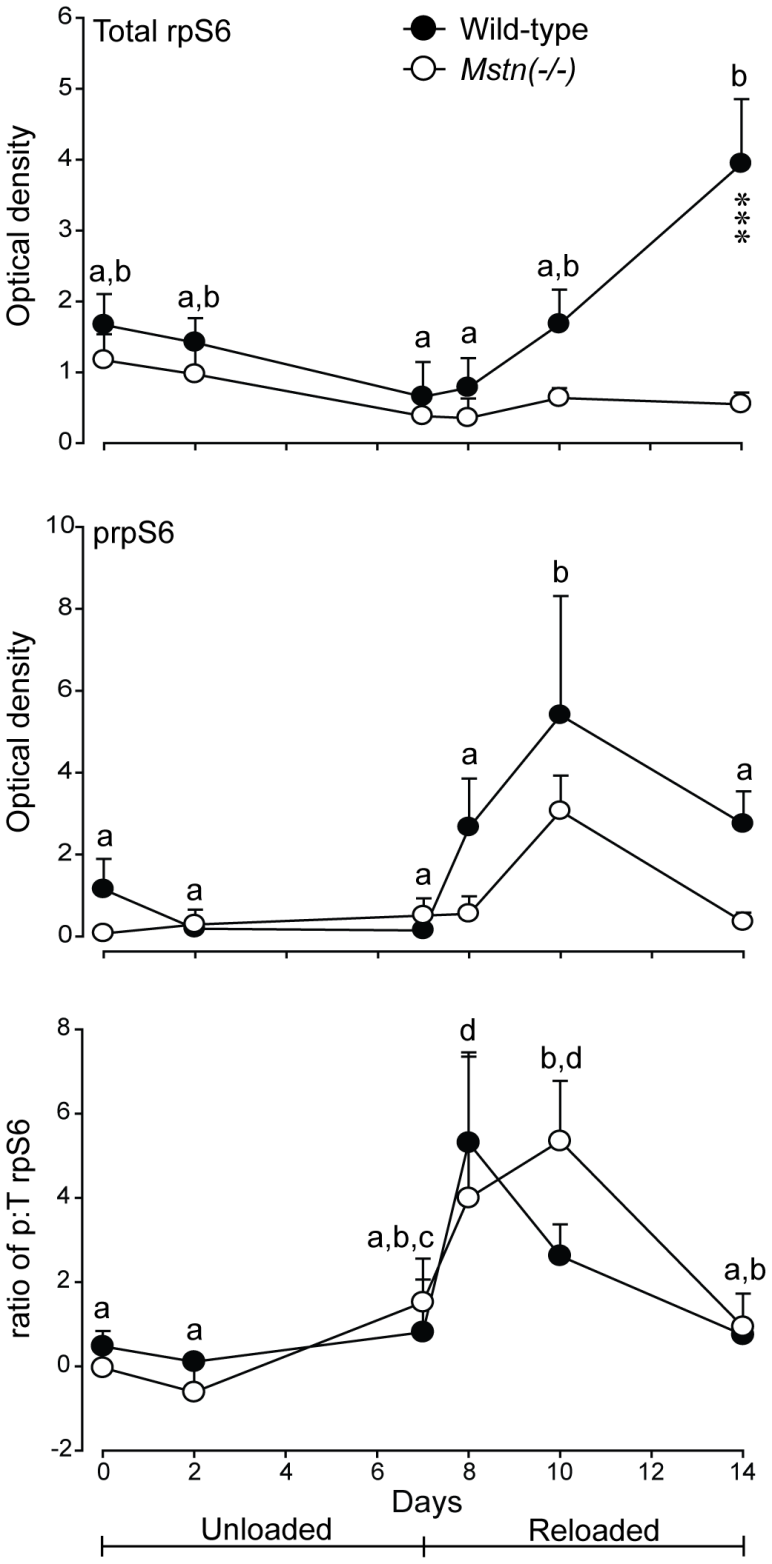
Abundance (mean+sem) of total (T) rpS6, phosphorylated (p) rpS6 and the ratio of p:T rpS6 in *gastrocnemius* muscles of *Mstn*(−/−) and wild-type mice (n = 6 per genotype and day) during seven days of unloading and seven days of reloading. Asterisks denote significant differences between genotypes at the days indicated (***P<0.001). Unlike letters denote significant differences (*P*<0.05) across days (independent of genotype).

The abundance of total rpS6 was greater in muscles of wild-type mice at d14 only (P<0.05). The pattern of phosphorylated rpS6 was not different between the two genotypes and was increased after one day of reloading (d8), then declined to pre-HS phosphorylated values at d14 (Figure 10). The ratio of phosphorylated to total rpS6 did not differ between genotypes and increased (*P*<0.05) on reloading to d10 and declined thereafter, in both genotypes (Figure 10).

**Figure 11:**
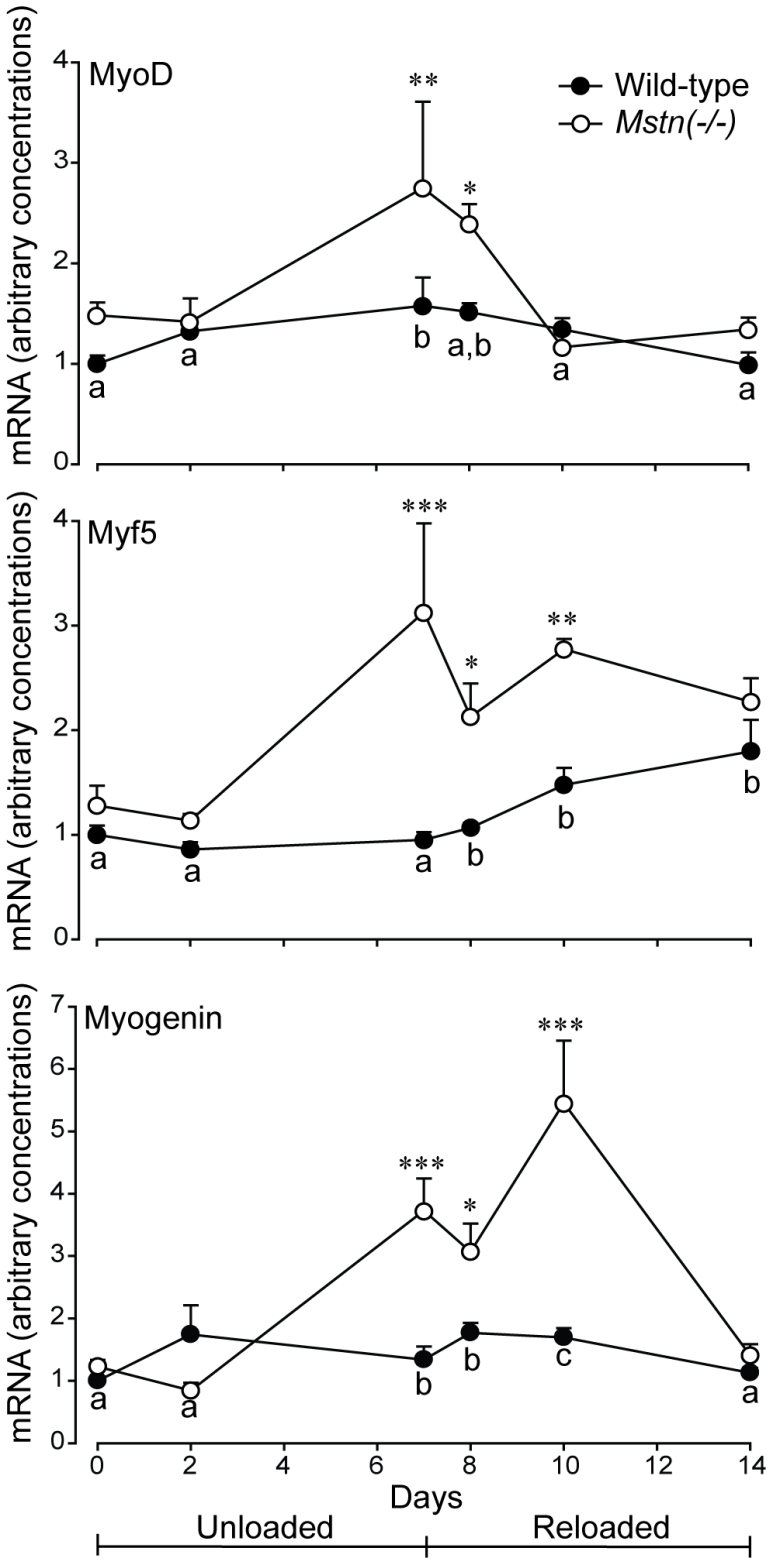
Arbitrary concentrations (mean+sem) of MyoD, Myf5 and Myogenin mRNA in the *B. femoris* muscles of *Mstn*(−/−) and wild-type mice during seven days of unloading and seven days of reloading (n = 6 per genotype and day). Asterisks denote differences between genotypes on days shown (*P<0.05, **P<0.01 and ***P<0.001). Unlike letters denote significant differences (*P*<0.05) across days (independent of genotype).

The concentrations of MyoD, Myf5 and myogenin mRNA increased progressively in *B. femoris* muscles of *Mstn*(−/−) mice during HS and remained elevated during the first three days of reloading before returning to the pre-HS state. In contrast, the expression of these genes did not change in the *B. femoris* muscles of wild-type controls during HS or reloading (Figure 11).

## Discussion

The current data confirm our previous report that *Mstn*(−/−) mice are more susceptible to muscle atrophy than wild-type mice during HS [12]. We now show that despite a greater loss of muscle mass during unloading, the wet mass and cross-sectional area of myofibres has largely recovered in *Mstn*(−/−) mice after seven days of reloading. We also confirm the particular susceptibility of type I and IIa MyHC-expressing soleus muscle to HS-induced atrophy and show that this is the case irrespective of a deficiency of myostatin [3], [62]. In addition, we had previously postulated that the type IIb MyHC-expressing myofibres were more susceptible to HS-induced atrophy in *Mstn*(−/−) mice [12]. Our data are consistent with *Mstn*(−/−) mice having a higher proportion of type IIb MyHC myofibres [41], [53]. We now show that the abundance of type IIb MyHC protein is lost and regained to a greater extent in muscles of *Mstn*(−/−) than of wild-type mice during unloading and reloading, respectively.

The mass of the *B. femoris* and *Quad* muscles of *Mstn*(−/−) mice had not fully recovered after 7 d of reloading. In addition, the mass of the Quad declined again from d10 to d14 of reloading, which may be attributed edema after reloading [63]. Previous studies have shown that the ability to recover muscle mass after unloading-induce atrophy differs with the age of mice. During the first month of post-natal life, recovery is greatest, but incomplete, possibly due to the need for satellite cells to provide nuclei to support the rapid post-natal phase of growth in young mammals (reviewed in [2]). Recovery is complete in young adults (3 to 4 months) because skeletal muscles have acquired their mature size and the number of myonuclei required for maintenance has been attained and is not lost during HS [64]. However, the muscles of elderly rodents (25 to 30 months) require longer to recover lost mass [65] and may require stimulation of protein synthetic pathways (via IGF-1) and recruitment of satellite cells for full recovery [66]. We speculate that the mass of the *B. femoris* and *Quad* muscles in *Mstn*(−/−) mice would be fully restored with a further week of reloading.

Apart from the soleus muscle, the wet mass of the other skeletal muscles of wild-type mice were not significantly reduced after 7d unloading. Although a longer period of unloading may have resulted in a greater loss of mass of muscles in both genotypes, our duration of unloading and reloading was sufficient to demonstrate the differences between the genotypes and prevented further ethical or potentially confounding health issues associated with excessive wasting in the *Mstn*(−/−) mice. Others have reported that atrophy of the soleus muscles of rats is associated with a reduction in the rate of protein synthesis within hours of HS [67]. An initially rapid (within 7 days) and then progressive decline in the abundance of actin and myosin ensues [3], [5], [68], [69]. Therefore, we opted to unload muscles for one week to capture the early signalling events leading to subsequent atrophy of skeletal muscles. Despite the lack of change in the wet mass of skeletal muscles of wild-type mice, we did observe a decrease in the abundance of MyHC IIb protein in *B. femoris* and a decrease in the cross-sectional area of *gastrocnemius* muscle fibers of wild-type mice which suggests that atrophy had begun.

The ubiquitin-proteasome ligases, MAFbx and MuRF1, have been shown to be increased during numerous conditions, including HS, which result in atrophy of skeletal muscle in rodents [27], [29], [70]–[73]. Consistent with this observation, the concentrations of mRNA of both ligases increased during HS, but were increased to a greater extent in muscles of *Mstn*(−/−) than in wild-type mice. However, the increased concentrations peaked at day 2, while HS continued to day 7, which suggests that other processes are involved in progressing atrophy. In addition, concentrations of MUSA1 were increased to a similar extent in both genotypes at day 2 of unloading, which supports a role for this BMP-regulated ubiquitin ligase in regulating atrophy of skeletal muscle [31]. However, our data suggest that the greater atrophy in skeletal muscles of *Mstn*(−/−) mice can be explained by a greater upregulation of MAFbx and MuRF1, rather than MUSA1, compared with muscles of wild-type mice. If myostatin activates these ligases [32], we would have expected the concentrations to have been unchanged in *Mstn*(−/−) mice, but this was not the case. In contrast, others have shown that myostatin inhibits MAFbx and MuRF1 mRNA in cultured myotubes [25]. Therefore, our data suggest that the relationship between the activation of these ligases and an increase in myostatin is not causal in skeletal muscle. The lack of difference in the concentrations of the three ligases between *Mstn*(−/−) and wild-type muscles during reloading are further evidence of the independence of myostatin and the ubiquitin-proteasome system.

The autophagy-lysosome system was also upregulated with a greater expression of LC3b, Gabarapl1 and Atg4b in the *B. femoris* muscles of *Mstn*(−/−) mice, at least during the first two days of unloading. Myostatin has been shown to induce transcription of autophagy-lysosomal genes in myotubes from mice and trout [74], [75]. Again, however, our data suggest that activation is not myostatin-dependent given that activation was greater, at least for LC3b, in muscles of *Mstn*(−/−) compared with wild-type mice. Therefore, we suggest that the autosomal-lysosomal system is upregulated in conjunction with the ubiquitin-proteasome system to help clear myofibrillar proteins, cellular debris and organelles. Activation of the autophagy-lysosomal system is also important during regrowth of muscle. Reloading after HS is associated with an influx of inflammatory-response cells (macrophages, neutrophils, mast cells) [76]–[78] and, therefore, the increased expression of Atg4b and LC3b during reloading is consistent with removal of aberrant proteins and organelles produced during the recovery phase.

A key finding of the current study was that the abundance of 4E-BP1 was greater in muscles of *Mstn*(−/−) mice, compared with those of wild-type mice. As a result, the ratio of phosphorylated to total 4E-BP1 was lower in muscles of *Mstn*(−/−) compared with wild-type mice before HS, which suggests impaired initiation of translation. Our data agree with the early decline in the synthesis of protein during unloading [67]. However, our data are at odds with those of others who showed that there was no difference in the abundance of total 4E-BP1 in muscles of wild-type mice after injecting an antibody to block myostatin, which resulted in an increased rate of protein synthesis [26]. The difference may be explained by the transient effect of an antibody to block myostatin in the former study as compared with the constitutive absence of myostatin in the transgenic animals used here. Our data are in agreement with the greater amount of 4E-BP1 bound to eIF4E (m^7^GTP-sepharose pull-down assay). Therefore, the greater abundance of 4E-BP1 in muscles of *Mstn*(−/−) mice was, likely, repressing the initiation of protein synthesis compared with muscles of wild-type mice. In addition, the ratio of phosphorylated to total 4E-BP1 was reduced in the *gastrocnemius* of both genotypes during HS, which supports previous studies that showed a decrease in protein synthesis during HS in rodents [3], [67]. The lower ratio of phosphorylated to total 4E-BP1 in muscles of *Mstn*(−/−) mice may compromise translation of mRNA to a greater extent than in wild-type mice and help to explain the greater loss of muscle mass during HS. Importantly, these changes were sustained for the seven days of HS, rather than the transient changes seen in the ubiquitin-proteasome and autophagy-lysosomal genes. Therefore, the greater loss of muscle mass in *Mstn*(−/−) may arise from an initial degradation of protein and a sustained decrease in translation. The increase in the ratio of phosphorylated to total eIF2α in muscles of both genotypes is also consistent with a reduced rate of translation during HS. The ratio of phosphorylated to total eIF2α was not restored to pre-HS levels during reloading to 14 days, suggesting that there was incomplete recovery of protein synthetic mechanisms.

We show here that the ratio of phosphorylated to total rpS6 was not different between genotypes and was not altered during HS, but did increase in both genotypes consistent with an increase in protein synthesis during reloading. These data appear to be at odds with the higher rate of protein synthesis in *gastrocnemius* muscles of *Mstn*(−/−) mice under normal loading conditions and the action of myostatin to inhibit protein synthesis *in vitro* [79], [80]. Furthermore, others have observed a higher RNA:DNA ratio compared with wild-type mice, but a lower concentration of DNA per microgram of tissue, suggesting that there is greater transcription and translation occurring with fewer nuclei per unit volume of muscle [79]. However, the fractional rate of synthesis was not different in *gastrocnemius* muscles of adult *Mstn*(−/−) compared with wild-type mice, which may point to a potential mechanism for the susceptibility of the muscles of *Mstn*(−/−) mice to atrophy during HS. Indeed, skeletal muscles of *Mstn*(−/−) mice are weaker (when corrected for cross-sectional area), fatigue faster and have smaller and weaker tendons and fewer mitochondria per unit volume of muscle [41], [81], [82]. Therefore, it is tenable that hypertrophy occurs during post-natal development in skeletal muscles of *Mstn*(−/−) mice with compromised translational machinery. However, when placed under a physiological stress, such as HS, *Mstn*(−/−) mice are unable to respond as readily as wild-type mice and are, therefore, more susceptible to atrophy. In support, others have recently shown that *Mstn*(−/−) mice lose more muscle mass during tumour-induced cachexia [83]. However, therapies targeting myostatin, or related proteins, can protect against muscle atrophy in mice [83]–[85]. Perhaps the increased RNA:DNA in muscles of *Mstn*(−/−) mice is a compensatory mechanism for the repression of eIF4E by 4E-BP1, which may compromise translation of mRNA.

While the activation of satellite cells has recently been shown to be associated with the recovery of muscle mass in adult rats after 14 days of HS [86], it is generally agreed that recovery and hypertrophy of skeletal muscle in adults does not require the recruitment of satellite cells [14], [15], [18], including after HS [16], [17]. The basal turnover of myonuclei of 1–2% per week in adult rat muscle is sufficient for maintaining adult muscle mass [87] and for regrowth. Therefore, the increased expression of MRFs observed in the skeletal muscles of adult *Mstn*(−/−) mice observed here probably does not reflect the recruitment of satellite cells, although we cannot rule out this possibility without further evidence. Pertinent to this study though, MRFs have been reported to contribute to the activation of atrogenes [88] and in maintaining the balance in myofibre types [89]. The E-boxes, to which MRFs bind, have been shown to be present in the promoter regions of MyHC genes and atrogenes [88], [90]. MyoD is preferentially expressed in fast-twitch muscle fibres (type IIx and IIb), while myogenin is preferentially expressed in slow-twitch (type I) muscle fibres [50], [51]. In support, Ekmark et al [89] proposed that myofibre type is regulated by a balance between MyoD and myogenin. In contrast, others have shown that the expression of MyoD and myogenin is increased in skeletal muscles during HS [52], [91] and after denervation [88]. Furthermore, myogenin, but not MyoD, increases MAFbx and MuRF1 mRNA when expressed in myoblasts [88], which partially accords with the increased expression of atrogenes in the current study. Importantly, expression of MRFs and atrogenes was greatly reduced in muscles of myogenin-null mice that were denervated to induce atrophy [88]. However, our data do not support a role for myogenin to induce MAFbx and MuRF1 during HS because expression of the latter two genes was increased in both genotypes and peaked at d2 of HS, while expression of myogenin peaked at d8 in muscles of *Mstn*(−/−) mice and did not change in muscles of wild-type mice. Therefore, we suggest that the increased expression of MyoD mRNA may be protecting the higher proportion of type IIb MyHC in *Mstn*(−/−) mice. The role of Myf5 in adult skeletal muscle is not as clear, but given the pattern of expression, it is, perhaps, similar to that of MyoD.

We conclude that skeletal muscles of *Mstn*(−/−) mice are more susceptible to HS-induced atrophy than those of wild-type mice, but do recover on reloading. We also show that the higher proportion of type IIb MyHC in muscles of *Mstn*(−/−) mice accords with the greater susceptibility of those myofibres to atrophy during unloading. We speculate that a combination of a transient increase in the degradation of protein via the ubiquitin-proteasome and autophagy-lysosomal systems along with a sustained reduction of protein synthetic mechanisms (particularly that of greater 4E-BP1) underlie the susceptibility of muscles of *Mstn*(−/−) mice to unloading-induced atrophy. Increased expression of myogenin and MyoD may be protecting myofibres containing a higher proportion of type IIb MyHC protein in muscles of *Mstn*(−/−) mice. The reversal of these mechanisms may contribute to the faster recovery of muscle mass in *Mstn*(−/−) mice during reloading.

From these findings, we propose that antagonists to myostatin may not be a beneficial therapy during the atrophying phase of skeletal muscle, but may be beneficial during the recovery phase, where muscle is actively loaded. Furthermore, our data suggests that myostatin might be a therapy to administer during unloading-induced atrophy to protect the fast-twitch fibres in particular - a view that is perhaps contentious and departs from current dogma [7]. Despite this comment, others have shown that administration of the soluble activin receptor 2B reduces the extent of muscle atrophy during cachexia, which, perhaps, highlights the fact that other TGF-β members are activated in different pathological conditions to induced atrophy [83].
